# Osteoporotic Hip Fractures: Current Issues and Evolving Concepts of Surgical Management

**DOI:** 10.1155/2013/793138

**Published:** 2013-03-06

**Authors:** Frankie Leung, Fan Liu, Chris Morrey, Chang-Wug Oh

**Affiliations:** ^1^Department of Orthopaedics & Traumatology, The University of Hong Kong, Hong Kong; ^2^Department of Orthopaedics, Affiliated Hospital of NanTong University, NanTong, Jiangsu 226001, China; ^3^Department of Orthopaedic, Cairns Orthopaedic Clinic, Cairns Base Hospital, Cairns, QLD 4870, Australia; ^4^Department of Orthopedic Surgery, Kyungpook National University Hospital, Daegu 700-721, Republic of Korea

Osteoporotic hip fractures are the most severe kind of fragility fracture, with high risks of increased disability and mortality. Recent advances on surgical treatment have shed new light in the management of these fractures. Nevertheless, the poor bone quality sometimes renders the treatment unsuccessful and there remains a definite complication rate in its treatment. Up till now, there has been a continuous effort in improving the currently used implants and surgical techniques, ranging from joint replacements to various forms of internal fixation. In order to provide better care for these patients, there is an increasing need for better understanding of the factors responsible for determining a successful surgical outcome.

This special issue covers the important topic of osteoporotic hip fractures. Failure of management may be caused by a delay in diagnosis leading to delayed treatment, as highlighted by the two articles by S. Gill et al. and J. Harding et al. Since the fracture may occur with relatively small energy of trauma, a certain percentage of these injuries may not be clearly visible on radiographs and special imaging is required to make an early diagnosis. In this issue, J. Harding et al. reported on the use of Bristol hip view radiographs while S. Gill et al. suggested the use of multislice CT which had the same effectiveness as MRI.

Failure of management may also mean a poor choice of surgical fixation device. This is particularly common when surgeons need to find the best device for intertrochanteric fractures. Various methods have been used to investigate the effectiveness of different devices. S. Zhang et al. used three-dimensional computational modeling to check the geometrical fit of these implants in femur.R. Tao et al. compared the clinical outcomes of two fixation devices and X. Huang et al. reported on the meta-analysis of randomized comparative trials that used the two most commonly used devices. 

To complicate the issue even more, it was found recently that some fractures are related to the bisphosphonates that the patients take as prophylaxis to osteoporotic fractures. Some of these atypical fractures are incomplete and C. W. Oh et al. reported the effectiveness of intramedullary nailing in the treatment of these fractures.

In summary, osteoporotic hip fractures are a big burden to modern health care system. Further efforts should be dedicated to seek the best treatment options to become available for these patients. 

*Frankie Leung* *Frankie Leung* *Fan Liu* *Fan Liu* *Chris Morrey* *Chris Morrey* *Chang-Wug Oh* *Chang-Wug Oh*

